# Supplementary material for: Genetic Regulation of Unsaturated Fatty Acid Composition in C. elegans
Source: PLoS Genet. 2006 Jul 14;2(7):e108. doi: 10.1371/journal.pgen.0020108 (PMC1500810; doi:10.1371/journal.pgen.0020108)
Supplement: Supplementary file 1 [file pgen.0020108.st001.doc]

**Supplementary Table 1**. Sequence of DNA primers used in these studies.

| I. PRIMERS USED FOR DETECTION OF DELETION AND POINT MUTATIONS | | | |
| --- | --- | --- | --- |
|  |  |  |  |
| **name** | **gene** | **cosmid** | **primer sequence** |
| TB5 | fat-6 | VZK822L.1 | GGATTTGCTTCCAGGCAGGT |
| TB6 | fat-6 | VZK822L.1 | CCAGAGACGCAATATCTCGC |
| TB23 | fat-5 | W06D12.3 | TTGCCTCCGGCAAACAGACT |
| TB24 | fat-5 | W06D12.3 | ATTCTCAGGCTTGAGCTCAG |
| TB106 | fat-7 | F10D2.9 | GACCAGTGGGAAATAGTGCCTGAA |
| TB107 | fat-7 | F10D2.9 | GGCTTCTTGTGCGTAAGCATCAAC |
| TB108 | fat-7 | F10D2.9 | GGCTTCTTGTGCGTAAGCATCGAT |
|  |  |  |  |
| II. PRIMERS USED FOR QPCR EXPERIMENTS | | | |
|  |  |  |  |
| **name** | **gene** | **cosmid** | **primer sequence** |
| TB53 | fat-6 | VZK822L.1 | TCTACCAGCTCATCTTCGAGGC |
| TB54 | fat-6 | VZK822L.1 | GATCACGAGCCCATTCGATGAC |
| TB55 | fat-5 | W06D12.3 | CGATTTGTACGAGGATCCGGTG |
| TB56 | fat-5 | W06D12.3 | CAGTGGGAGACACTGTTGATGC |
| TB63 | fat-7 | F10D2.9 | GGAAGGAGACAGCATTCATTGCG |
| TB64 | fat-7 | F10D2.9 | GTCTTGTGGGAATGTGTGGTGG |
| TB57 | act-5 | T25C8.2 | TCAGGGAGTGATGGTCGGTATG |
| TB58 | act-5 | T25C8.2 | CTCGGTAAGGAGAACTGGGTGT |
| TB59 | tbb-2 | C36E8.5 | GAGGAGGAACCGGATCTGGAAT |
| TB60 | tbb-2 | C36E8.5 | GGCCTCGTTGTCAATGCAGTAG |
| TB61 | ubc-2 | M7.1 | AGGGAGGTGTCTTCTTCCTCAC |
| TB62 | ubc-2 | M7.1 | CGGATTTGGATCACAGAGCAGC |
| TB134 | ech-1 | C29F3.1 | GATATAGCCTCTGTATTGGTC |
| TB135 | ech-1 | C29F3.1 | AATTGTCCTCCACTCTTCGC |
| TB136 | acs-2 | F28F8.2 | AGTGAGACTTGACAGTTCCG |
| TB137 | acs-2 | F28F8.2 | CTTGTAAGAGAGGAATGGCTC |
|  |  |  |  |
| III. PRIMERS USED FOR GFP FUSION PROTEIN CONSTRUCTS | | | |
|  |  |  |  |
| **name** | **gene** | **cosmid** | **primer sequence** |
| TB36 | fat-5 | W06D12.3 | AAGTTGCCGGAAATCAAGTG |
| TB37 | fat-5 | W06D12.3 | CCATGAGAGGGTGGCTTTGTA |
| TB38 | fat-6 | VZK822L.1 | ACCATCTTCATCAATCTTGC |
| TB45 | fat-6 | VZK822L.1 | CACGTTACGCCACACAATTTC |
| TB43 | fat-7 | F10D2.9 | GAGCCGAATATGCACAGAAA |
| TB40 | fat-7 | F10D2.9 | TCTTGATTCTTCACTTCCGTG |
